# Supplementary material for: Assessment of the integrity of real-time electronic health record data used in clinical research
Source: PLoS One. 2026 Jan 9;21(1):e0340287. doi: 10.1371/journal.pone.0340287 (PMC12788664; doi:10.1371/journal.pone.0340287)
Supplement: S1 Table — (DOCX) [file pone.0340287.s001.docx]

**S1 Table**. **Statistics on number of patients and associated encounters for daily snapshots between April 12 to May 31, 2025.**

| Date of snapshot | Unique number of patients | Number of encounters | Number of IP encounter | Number of OP encounter |
| --- | --- | --- | --- | --- |
| 4/12/25 | 2,398,207 | 134,385,737 | 994,405 | 31,550,816 |
| 4/13/25 | 2,399,574 | 134,490,289 | 994,847 | 31,579,662 |
| 4/14/25 | 2,400,059 | 134,488,913 | 995,246 | 31,604,953 |
| 4/15/25 | 2,400,569 | 134,657,047 | 995,664 | 31,627,014 |
| 4/16/25 | 2,400,583 | 134,751,776 | 996,070 | 31,652,616 |
| 4/17/25 | 2,400,620 | 134,840,749 | 996,434 | 31,674,596 |
| 4/18/25 | 2,400,889 | 134,873,294 | 996,710 | 31,678,815 |
| 4/19/25 | 2,401,040 | 134,891,516 | 997,011 | 31,684,796 |
| 4/20/25 | 2,401,835 | 134,994,300 | 997,465 | 31,711,026 |
| 4/21/25 | 2,402,438 | 135,187,834 | 997,837 | 31,734,565 |
| 4/22/25 | 2,403,002 | 135,271,445 | 998,241 | 31,757,159 |
| 4/23/25 | 2,403,399 | 135,356,720 | 998,689 | 31,781,593 |
| 4/24/25 | 2,403,844 | 135,511,526 | 999,081 | 31,803,083 |
| 4/25/25 | 2,403,816 | 135,684,307 | 999,381 | 31,806,043 |
| 4/26/25 | 2,404,062 | 135,698,250 | 999,700 | 31,807,664 |
| 4/27/25 | 2,404,662 | 135,762,778 | 1,000,142 | 31,834,441 |
| 4/28/25 | 2,405,185 | 135,868,339 | 1,000,569 | 31,856,672 |
| 4/29/25 | 2,405,709 | 135,928,503 | 1,000,937 | 31,878,146 |
| 4/30/25 | 2,406,008 | 136,079,372 | 1,001,361 | 31,899,149 |
| 5/1/25 | 2,406,381 | 136,163,958 | 1,001,713 | 31,917,657 |
| 5/2/25 | 2,406,490 | 136,191,322 | 1,001,988 | 31,920,722 |
| 5/3/25 | 2,406,695 | 136,207,369 | 1,002,278 | 31,923,372 |
