# Supplementary material for: Assessment of the integrity of real-time electronic health record data used in clinical research
Source: PLoS One. 2026 Jan 9;21(1):e0340287. doi: 10.1371/journal.pone.0340287 (PMC12788664; doi:10.1371/journal.pone.0340287)
Supplement: S2 Table — (DOCX) [file pone.0340287.s002.docx]

**S2 Table**. **Number of patients with EHR clinical actions taken between consecutive daily snapshots from April 12, 2025, to May 3, 2025.**

| Date of snapshot | Add New Patient (AD) | Update Demographics (IR) | Update Patient ID (IC) | Delete Patient (DL) | Merge Patients (DM) |
| --- | --- | --- | --- | --- | --- |
| 4/12/25 | 0 | 0 | 0 | 0 | 0 |
| 4/13/25 | 1996 | 182 | 630 | 60 | 124 |
| 4/14/25 | 1178 | 168 | 672 | 76 | 62 |
| 4/15/25 | 1272 | 696 | 562 | 191 | 100 |
| 4/16/25 | 940 | 637 | 744 | 169 | 94 |
| 4/17/25 | 934 | 712 | 690 | 201 | 138 |
| 4/18/25 | 618 | 285 | 379 | 39 | 58 |
| 4/19/25 | 486 | 297 | 401 | 38 | 66 |
| 4/20/25 | 1055 | 653 | 513 | 84 | 99 |
| 4/21/25 | 967 | 998 | 511 | 215 | 51 |
| 4/22/25 | 950 | 1275 | 488 | 297 | 45 |
| 4/23/25 | 912 | 1738 | 515 | 410 | 70 |
| 4/24/25 | 805 | 1599 | 496 | 382 | 86 |
| 4/25/25 | 408 | 642 | 311 | 63 | 32 |
| 4/26/25 | 393 | 161 | 238 | 31 | 75 |
| 4/27/25 | 899 | 1587 | 534 | 346 | 66 |
| 4/28/25 | 708 | 1703 | 484 | 347 | 61 |
| 4/29/25 | 741 | 2371 | 462 | 558 | 51 |
| 4/30/25 | 803 | 332 | 432 | 83 | 75 |
| 5/1/25 | 817 | 473 | 435 | 118 | 156 |
| 5/2/25 | 460 | 204 | 283 | 42 | 75 |
| 5/3/25 | 383 | 179 | 275 | 23 | 22 |
