# Supplementary material for: Assessment of the integrity of real-time electronic health record data used in clinical research
Source: PLoS One. 2026 Jan 9;21(1):e0340287. doi: 10.1371/journal.pone.0340287 (PMC12788664; doi:10.1371/journal.pone.0340287)
Supplement: S3 Table — (DOCX) [file pone.0340287.s003.docx]

**S3 Table. Number of patients with EHR demographics change performed between consecutive daily snapshots from April 12, 2025, to May 3, 2025.**

| Date of Snapshot | Sex Change | DOB Change | Race Change | Ethnicity Change |
| --- | --- | --- | --- | --- |
| 4/12/25 | 0 | 0 | 0 | 0 |
| 4/13/25 | 8 | 50 | 516 | 133 |
| 4/14/25 | 6 | 24 | 583 | 136 |
| 4/15/25 | 13 | 39 | 452 | 129 |
| 4/16/25 | 6 | 49 | 614 | 152 |
| 4/17/25 | 15 | 40 | 569 | 144 |
| 4/18/25 | 5 | 39 | 301 | 80 |
| 4/19/25 | 4 | 31 | 343 | 66 |
| 4/20/25 | 4 | 15 | 438 | 119 |
| 4/21/25 | 13 | 14 | 437 | 125 |
| 4/22/25 | 11 | 8 | 419 | 121 |
| 4/23/25 | 6 | 19 | 448 | 102 |
| 4/24/25 | 8 | 13 | 411 | 122 |
| 4/25/25 | 4 | 11 | 259 | 74 |
| 4/26/25 | 1 | 4 | 213 | 50 |
| 4/27/25 | 3 | 12 | 444 | 128 |
| 4/28/25 | 4 | 12 | 411 | 128 |
| 4/29/25 | 9 | 7 | 384 | 115 |
| 4/30/25 | 3 | 17 | 362 | 107 |
| 5/1/25 | 7 | 14 | 361 | 102 |
| 5/2/25 | 4 | 19 | 242 | 46 |
| 5/3/25 | 2 | 13 | 233 | 56 |
