# Supplementary material for: Assessment of the integrity of real-time electronic health record data used in clinical research
Source: PLoS One. 2026 Jan 9;21(1):e0340287. doi: 10.1371/journal.pone.0340287 (PMC12788664; doi:10.1371/journal.pone.0340287)
Supplement: S4 Table — (DOCX) [file pone.0340287.s004.docx]

**S4 Table. Number of patients with inpatient encounters whose discharge information was changed from that in the baseline snapshot of April 12, 2025.**

| Date of Snapshot | Discharge Status Change Only | Discharge Time Change Only | Both |
| --- | --- | --- | --- |
| 4/12/25 | 0 | 0 | 0 |
| 4/13/25 | 359 | 23 | 0 |
| 4/14/25 | 422 | 43 | 3 |
| 4/15/25 | 440 | 45 | 5 |
| 4/16/25 | 449 | 48 | 6 |
| 4/17/25 | 479 | 69 | 8 |
| 4/18/25 | 479 | 70 | 8 |
| 4/19/25 | 478 | 70 | 9 |
| 4/20/25 | 490 | 87 | 9 |
| 4/21/25 | 494 | 146 | 9 |
| 4/22/25 | 501 | 163 | 9 |
| 4/23/25 | 503 | 163 | 9 |
| 4/24/25 | 504 | 205 | 9 |
| 4/25/25 | 505 | 205 | 9 |
| 4/26/25 | 505 | 205 | 9 |
| 4/27/25 | 506 | 206 | 9 |
| 4/28/25 | 508 | 228 | 9 |
| 4/29/25 | 511 | 243 | 10 |
| 4/30/25 | 511 | 260 | 10 |
| 5/1/25 | 514 | 287 | 10 |
| 5/2/25 | 516 | 287 | 10 |
| 5/3/25 | 517 | 287 | 10 |
