# Supplementary material for: Assessment of the integrity of real-time electronic health record data used in clinical research
Source: PLoS One. 2026 Jan 9;21(1):e0340287. doi: 10.1371/journal.pone.0340287 (PMC12788664; doi:10.1371/journal.pone.0340287)
Supplement: S5 Table — Number of patients with outpatient encounters whose discharge information was changed from that in the baseline snapshot of April 12, 2025. (DOCX) [file pone.0340287.s005.docx]

**S5 Table. Number of patients with outpatient encounters whose discharge information was changed from that in the baseline snapshot of April 12, 2025.**

| Date of Snapshot | Discharge Status Change Only | Discharge Time Change Only | Both |
| --- | --- | --- | --- |
| 4/12/25 | 0 | 0 | 0 |
| 4/13/25 | 872 | 561 | 11 |
| 4/14/25 | 1240 | 937 | 36 |
| 4/15/25 | 1366 | 1003 | 49 |
| 4/16/25 | 1483 | 1046 | 54 |
| 4/17/25 | 1521 | 1050 | 60 |
| 4/18/25 | 1549 | 1048 | 66 |
| 4/19/25 | 1565 | 1048 | 67 |
| 4/20/25 | 1594 | 1049 | 72 |
| 4/21/25 | 1615 | 1048 | 79 |
| 4/22/25 | 1649 | 1049 | 82 |
| 4/23/25 | 1690 | 1050 | 82 |
| 4/24/25 | 1754 | 1050 | 82 |
| 4/25/25 | 1770 | 1050 | 82 |
| 4/26/25 | 1787 | 1050 | 84 |
| 4/27/25 | 1809 | 1050 | 84 |
| 4/28/25 | 1815 | 1050 | 85 |
| 4/29/25 | 1845 | 1050 | 86 |
| 4/30/25 | 1873 | 1049 | 91 |
| 5/1/25 | 2226 | 1049 | 91 |
| 5/2/25 | 6270 | 996 | 144 |
| 5/3/25 | 6484 | 996 | 144 |
