# Supplementary material for: Assessment of the integrity of real-time electronic health record data used in clinical research
Source: PLoS One. 2026 Jan 9;21(1):e0340287. doi: 10.1371/journal.pone.0340287 (PMC12788664; doi:10.1371/journal.pone.0340287)
Supplement: Attachment — Submitted filename: EHRQuality_ResponsetoReviewers.docx [file pone.0340287.s007.docx]

Reviewers' comments:

Reviewer's Responses to Questions

**Comments to the Author**

1. Is the manuscript technically sound, and do the data support the conclusions?

The manuscript must describe a technically sound piece of scientific research with data that supports the conclusions. Experiments must have been conducted rigorously, with appropriate controls, replication, and sample sizes. The conclusions must be drawn appropriately based on the data presented.

Reviewer #1: No

2. Has the statistical analysis been performed appropriately and rigorously?

Reviewer #1: I Don't Know

3. Have the authors made all data underlying the findings in their manuscript fully available?

The [PLOS Data policy](http://track.editorialmanager.com/CL0/http:%2F%2Fwww.plosone.org%2Fstatic%2Fpolicies.action%23sharing/1/010f019a173442d9-397e3a2c-d556-4f39-b0dc-552fc47f7be0-000000/LhTdORQR6mXSZjVtfDsaDyKUe7PCrcqNROHx8p1AME8=234) requires authors to make all data underlying the findings described in their manuscript fully available without restriction, with rare exception (please refer to the Data Availability Statement in the manuscript PDF file). The data should be provided as part of the manuscript or its supporting information, or deposited to a public repository. For example, in addition to summary statistics, the data points behind means, medians and variance measures should be available. If there are restrictions on publicly sharing data—e.g. participant privacy or use of data from a third party—those must be specified.

Reviewer #1: No

4. Is the manuscript presented in an intelligible fashion and written in standard English?

PLOS ONE does not copyedit accepted manuscripts, so the language in submitted articles must be clear, correct, and unambiguous. Any typographical or grammatical errors should be corrected at revision, so please note any specific errors here.

Reviewer #1: Yes

5. Review Comments to the Author

Please use the space provided to explain your answers to the questions above. You may also include additional comments for the author, including concerns about dual publication, research ethics, or publication ethics. (Please upload your review as an attachment if it exceeds 20,000 characters)

**Reviewer #1:** Liu and colleagues report their analysis of the integrity of real-time electronic health records (EHR) data for use in clinical research, in which they assess the stability and completeness of the recorded information in the Yale New Haven Health (YNHH) clinical data warehouse and conclude that a time lag of 4-7 days are required for records to stabilise to ensure validity for research. The work is interesting and has merit to other researchers who can utilise the framework in assessing the integrity of other local EHR systems, however I do feel the findings presented are likely to be very specific to the YNHH context, and thus the external validity of the results is uncertain.

Major comments

1. The methods section would benefit from further description of the YNHH dataset, e.g. patients that are included, sector of healthcare etc. How records enter the YNHH dataset, does each clinical encounter generate a new record, who inputs the data, etc?

The YNHH dataset was populated in real-time, as the YNHH CHP platform maintains a daily-updated data pipeline with clinical data extracts sourced from the YNHH EHR system. We have updated the first paragraph of the methods section to clarify this.

2. The methods section would benefit from revision to make it clearer which samples of data used are in the analysis, currently the methods refers only to data between April/May 2025, however the axes of figures 3-5 shows data spanning July/Nov 2024, and results section also describes additional methods relating to data in November 2024. It’s not fully clear to me why the dates would change for these different assessments so this should be spelled out. It would also be helpful to know if there was any rationale for the choice of baseline dates selected and why 26 snapshots were used?

We thank the reviewer for pointing out that clarification was needed on why daily snapshots were taken between April/May 2025, while the weekly snapshots were taken between July/Nov 2024. The weekly snapshots taken between July/November 2024 were used for Figures 3 and 4 because they reflect the most complete and temporally continuous set of EHR snapshots available. To strengthen our analyses and determine whether the July/November 2024 trends observed were consistent across the year, we analyzed daily snapshots in April/May 2025. Recognizing that the Benchmark 3 analysis could be strengthened with the daily April/May 2025 data (previously using daily snapshots taken in November 2024), we have revised the Benchmark 3 results and Figure 5 with data from daily snapshots taken between April 12, 2025, and May 3, 2025. This update revealed a spike in the number of patients with outpatient encounters whose discharge status changed after the first of May, and we’ve discussed potential reasons for this in the Discussion. All daily snapshot data are now included in the Supplementary Information tables. We have also included the justifications for the daily and weekly date ranges in the Methods and Discussion sections, including specifying which analysis pertains to which statistics and figures. We have revised each of the figure legends to clarify the corresponding date range and snapshot frequency shown.

3. The results section is very descriptive and contains few statistical results, which are instead included in an appendix. I would strongly suggest adding more summary statistics to support assertions being made.

We agree that the Results section should be strengthened with explicit statistical results. For each benchmark in the Results section, we have now included summary statistics (median and IQR) in addition to providing each of the raw, daily patient counts for each category of EHR change in the clearly referenced Supplementary tables.

4. Conclusions “Our findings indicate that real-time EHR data require 4–7 days for discharge information stabilization and systematic monitoring of demographic field consistency to ensure research validity.” I would probably suggest revising this statement slightly to acknowledge that this is a finding within the YNHH dataset, as external validity is not proven.

While these findings were observed within the YNHH dataset, the data integrity challenges we identified are likely inherent to the OMOP transformation process rather than institution-specific EHR practices, suggesting these patterns should be replicable across healthcare systems using OMOP CDM. We have revised the Discussion section paragraph on limitations to acknowledge that external validity as a limitation of this study and to clarify that use of OMOP-standardized data implies generalizability to other institutions using the OMOP common data model.

Minor

5. The first benchmark assessment relates to ‘clinical actions such as additions, deletions, and merges’ – I would suggest defining exactly what ‘clinical actions’ means as it is not entirely clear to me and I’m assuming it refers to specific clinical events or healthcare encounters than have happened, and the additions, deletions or merges occur if an update has been made to either add a new event or more accurately reflect one which has taken place?

Yes, “clinical actions” refers to database-level changes in patient records between snapshots. These include both prospective additions (new patients receiving care, new clinical encounters) and retrospective modifications (corrections to patient identifiers, merging of duplicate records, updates to demographic information, or deletion of erroneous entries). These changes can occur as clinical care is delivered and as administrative staff continuously refine data accuracy in the EHR system. We have revised the Methods section to clarify this terminology.

6. Race and ethnicity data were shown to be frequently updated, do you have any further insight into how race/ethnicity data are collected? Are they self-assigned or healthcare provider assigned categories? The latter tends be more prone to data quality issues therefore specifying this would be informative.

Race and ethnicity data at YNHH are self-assigned by patients during registration and clinical encounters. The frequent updates we observed could reflect several factors: (1) incomplete initial collection. For instance, fields left blank during emergency visits may be completed during subsequent scheduled encounters when more comprehensive patient intake occurs. (2) administrative staff may retrospectively update free-text entries to match structured OMOP vocabulary standards. (3) patients who initially declined to provide race/ethnicity information may later consent to share this data (4) patients seen across different YNHH facilities or departments may provide slightly different responses at each location, triggering reconciliation processes that update records to the most recent response. We appreciate your observation and have included this discussion in the Discussion section.

7. Careful review needed to check all acronyms spelled out at first use eg. BM-1 /consider if even needs to be abbreviated

We have revised so that all acronyms are spelled out at first usage.

8. Figures 3 and 4 – I am curious to understand if there is any explanations for the apparent spike in patient numbers on 09/27/24 ?

Interestingly, we observed spikes in patient numbers on Fridays in September and November 2024. This pattern may be attributable to a higher number of scheduled or emergent encounters on Fridays, delayed documentation finalization from earlier weekdays, or ETL pipeline scheduling that aggregates data captured throughout the week for Friday processing.

**Journal Requirements:**

1. When submitting your revision, we need you to address these additional requirements.

Please ensure that your manuscript meets PLOS ONE's style requirements, including those for file naming. The PLOS ONE style templates can be found at

<https://journals.plos.org/plosone/s/file?id=wjVg/PLOSOne_formatting_sample_main_body.pdf> and

<https://journals.plos.org/plosone/s/file?id=ba62/PLOSOne_formatting_sample_title_authors_affiliations.pdf>

2. Thank you for stating the following in the Competing Interests section:

Harlan Krumholz works under contract with the Centers for Medicare & Medicaid Services to support quality measurement programs; was a recipient of a research grant, through Yale, from Medtronic and the U.S. Food and Drug Administration to develop methods for post-market surveillance of medical devices; was a recipient of a research grant with Medtronic and is the recipient of a research grant from Johnson & Johnson, through Yale University, to support clinical trial data sharing; was a recipient of a research agreement, through Yale University, from the Shenzhen Center for Health Information for work to advance intelligent disease prevention and health promotion; collaborates with the National Center for Cardiovascular Diseases in Beijing; receives payment from the Arnold & Porter Law Firm for work related to the Sanofi clopidogrel litigation, from the Ben C. Martin Law Firm for work related to the Cook Celect IVC filter litigation, and from the Siegfried and Jensen Law Firm for work related to Vioxx litigation; chairs a Cardiac Scientific Advisory Board for UnitedHealth; was a participant/participant representative of the IBM Watson Health Life Sciences Board; is a member of the Advisory Board for Element Science, the Advisory Board for Facebook, and the Physician Advisory Board for Aetna; and is the co-founder of HugoHealth, a personal health information platform, and co-founder of Refactor Health, an AI-augmented data management platform for healthcare.

Wade Schulz is an investigator for a research agreement, through Yale University, from the Shenzhen Center for Health Information for work to advance intelligent disease prevention and health promotion; collaborates with the National Center for Cardiovascular Diseases in Beijing; is a technical consultant to HugoHealth, a personal health information platform, and cofounder of Refactor Health, an AI-augmented data management platform for healthcare; is a consultant for Interpace Diagnostics Group, a molecular diagnostics company.

Guannan Gong is the founder of CtrlTrial Inc., an AI-augmented patient screening platform for clinical trials.

We note that one or more of the authors are employed by a commercial company.

a. Please provide an amended Funding Statement declaring this commercial affiliation, as well as a statement regarding the Role of Funders in your study. If the funding organization did not play a role in the study design, data collection and analysis, decision to publish, or preparation of the manuscript and only provided financial support in the form of authors' salaries and/or research materials, please review your statements relating to the author contributions, and ensure you have specifically and accurately indicated the role(s) that these authors had in your study. You can update author roles in the Author Contributions section of the online submission form.

Please also include the following statement within your amended Funding Statement.

“The funder provided support in the form of salaries for authors, but did not have any additional role in the study design, data collection and analysis, decision to publish, or preparation of the manuscript. The specific roles of these authors are articulated in the ‘author contributions’ section.”

If your commercial affiliation did play a role in your study, please state and explain this role within your updated Funding Statement.

Thank you for your guidance regarding the Funding Statement and disclosure of commercial affiliations. We confirm that none of the commercial relationships were involved in any aspect of this research.

We have revised the Funding Statement to include the required text:

**“No commercial entity provided salary support, financial support, or research materials for this study, and no commercial entity played a role in the study design, data collection and analysis, decision to publish, or preparation of the manuscript. The specific roles of these authors are articulated in the ‘author contributions’ section.”**

Additionally, we will update the Author Contributions section in the online submission system to accurately reflect the roles of all authors as requested.

b. Please also provide an updated Competing Interests Statement declaring this commercial affiliation along with any other relevant declarations relating to employment, consultancy, patents, products in development, or marketed products, etc.

Within your Competing Interests Statement, please confirm that this commercial affiliation does not alter your adherence to all PLOS ONE policies on sharing data and materials by including the following statement: "This does not alter our adherence to  PLOS ONE policies on sharing data and materials.” (as detailed online in our guide for authors <http://journals.plos.org/plosone/s/competing-interests>) . If this adherence statement is not accurate and  there are restrictions on sharing of data and/or materials, please state these. Please note that we cannot proceed with consideration of your article until this information has been declared.

Please include both an updated Funding Statement and Competing Interests Statement in your cover letter. We will change the online submission form on your behalf.

Thank you for the additional guidance regarding the Competing Interests Statement. We confirm that we will provide an updated Competing Interests Statement declaring the relevant commercial affiliation, as well as any other required disclosures relating to employment or financial relationships.

The updated Competing Interests Statement will include the required language:

**“This does not alter our adherence to PLOS ONE policies on sharing data and materials.”**

We also confirm that there are **no restrictions on data or material sharing**, and the commercial affiliation did not influence the study design, data collection, analysis, decision to publish, or manuscript preparation.

As requested, we will include both the updated Funding Statement and the updated Competing Interests Statement in the cover letter. We appreciate that the editorial office will update the online submission form on our behalf.

3. Thank you for uploading your study's underlying data set. Unfortunately, the repository you have noted in your Data Availability statement does not qualify as an acceptable data repository according to PLOS's standards.

At this time, please upload the minimal data set necessary to replicate your study's findings to a stable, public repository (such as figshare or Dryad) and provide us with the relevant URLs, DOIs, or accession numbers that may be used to access these data. For a list of recommended repositories and additional information on PLOS standards for data deposition, please see <https://journals.plos.org/plosone/s/recommended-repositories>.

The original patient dataset is not publicly shareable due to containing PHI. We have added the weekly and daily patient counts analyzed for each benchmarking analysis in the Supplementary Information tables; these tables constitute all the shareable datasets for this study.

4. Please include captions for your Supporting Information files at the end of your manuscript, and update any in-text citations to match accordingly. Please see our Supporting Information guidelines for more information: <http://journals.plos.org/plosone/s/supporting-information>.

We have included captions for the Supporting Information tables at the end of the manuscript.

5. If the reviewer comments include a recommendation to cite specific previously published works, please review and evaluate these publications to determine whether they are relevant and should be cited. There is no requirement to cite these works unless the editor has indicated otherwise.

NA
