# Supplementary material for: Assessment of the integrity of real-time electronic health record data used in clinical research
Source: PLoS One. 2026 Jan 9;21(1):e0340287. doi: 10.1371/journal.pone.0340287 (PMC12788664; doi:10.1371/journal.pone.0340287)
Supplement: Attachment — Submitted filename: EHRQuality_ResponsetoReviewers_v2.docx [file pone.0340287.s008.docx]

Response to the reviewers

12/17/2025

*Assessment of the integrity of real-time electronic health record data used in clinical research*

Reviewer #1: Liu and colleagues have responded positively to all prior comments and the manuscript and its reporting is far strengthened by the revisions. One minor remaining comment I have relates to description of methods lingering the results section. For BM-2, it is suggested that this deeper analysis was conducted due the findings of BM-1. It would be helpful to state this earlier in the methods section, and ideally differentiate what analyses were pre-specified and which were post-hoc, which it sounds like BM-2 may have been.

We thank the reviewer for their continued feedback and support. We have updated the Methods section to explicitly categorize BM-1 and BM-3 as pre-specified analyses, while identifying BM-2 as a post-hoc investigation sparked by the demographic instability observed in BM-1. We also relocated the methodological rationale for BM-2 from the Results to the Methods to ensure a clear separation of study design and findings. The Results section now opens directly with the quantitative findings regarding demographic updates in 2024. These revisions enhance the manuscript's clarity and adhere to the distinction between planned and exploratory analyses.
